# Transplantation immunology: paradigm shift from systemic suppression to microenvironment remodeling and precision modulation

**DOI:** 10.3389/fcell.2026.1835561

**Published:** 2026-05-21

**Authors:** Siqi Huang, Shaochen Yu, Mengjie Zhang, Yuting Huang, Beibei Tian, Langlang Yang, Jian Lu

**Affiliations:** 1 Department of Oncology, Guilin Hospital of the Second Xiangya Hospital, Central South University, Guilin, Guangxi, China; 2 Department of Emergency and Critical Care Medicine, Chuzhou Integrated Traditional Chinese and Western Medicine Hospital, Chuzhou, Anhui, China; 3 Department of Gastroenterology, The First Affiliated Hospital of Anhui Medical University, Hefei, Anhui, China

**Keywords:** antibody-mediated rejection, engineered regulatory T cells, immune tolerance, local delivery, tissue-resident memory T cells (TRM), transplantation immunology, xenotransplantation

## Abstract

Organ transplantation remains the definitive therapy for end-stage organ failure, yet its long-term success is limited by allograft rejection. Conventional immunosuppressive regimens, while effective against acute rejection, are hampered by non-specific mechanisms that lead to serious complications such as infections and malignancies, posing a major barrier to long-term graft survival. Recent advances in high-dimensional technologies—including single-cell sequencing, epigenomics, and metabolomics—are driving a paradigm shift in transplant immunology from “broad suppression” toward “precision modulation.” This review systematically delineates the hierarchical network and regulatory mechanisms of the alloimmune response, with a focus on four pioneering therapeutic strategies: engineered cellular therapies, targeted biologics, local microenvironment remodeling, and metabolic/epigenetic interventions. It further discusses the integration of novel biomarkers, high-dimensional technologies, and artificial intelligence to establish a precision medicine framework for personalized immune management. Finally, future directions and challenges—including xenotransplantation, spatial multi-omics, and immune aging—are explored, providing a conceptual and practical roadmap for the fundamental transition from systemic immunosuppression to precision modulation of the immune microenvironment.

## 1 Introduction

Organ transplantation is the definitive treatment for end-stage organ failure, yet its long-term success remains constrained by the fundamental challenge of allograft rejection. Over the past three decades, conventional immunosuppressive protocols centered on calcineurin inhibitors have markedly reduced acute rejection rates. However, their non-specific mechanisms contribute to serious complications—including infections, malignancies, and metabolic disorders—that constitute a major bottleneck for long-term graft survival. These complications not only impair patients’ quality of life but also promote gradual graft dysfunction, with approximately 50% of kidney transplant recipients facing graft loss within 10 years post-transplantation (Karpen et al., 2021).

This clinical impasse has spurred a profound re-evaluation within the field, accelerating a paradigm shift from “broad immunosuppression” to “precision immunomodulation.” Advances in single-cell sequencing, epigenetic profiling, and metabolomics have deepened our understanding of the immune system to an unprecedented level (Bao et al., 2025; Xie et al., 2024). From the initial inflammatory cascade triggered by damage-associated molecular patterns to the long-term memory barriers formed by tissue-resident immune cells, each step reveals remarkable complexity and plasticity. In particular, insights into the immune microenvironment have uncovered intricate interaction networks between local immune and non-immune cells, providing a critical theoretical foundation for novel therapeutic strategies.

Against this backdrop, this review first outlines the hierarchical network and regulatory logic of the alloimmune response. It then highlights four key innovative strategies enabling the shift from “broad suppression” to “precision rebuilding.” Subsequently, it discusses the establishment of a precision medicine paradigm through novel biomarkers, high-dimensional technologies, and artificial intelligence. Finally, future challenges and directions are analyzed, charting a clear pathway for the fundamental transition from systemic suppression to microenvironment remodeling in transplant immunology.

## 2 Hierarchical network and regulatory mechanisms of the alloimmune response

### 2.1 Innate immune activation and microenvironment priming

The alloimmune response is initiated by a highly programmed amplification of “danger signals” orchestrated by the innate immune system. Its core trigger is ischemia-reperfusion injury (IRI), which represents an active immunogenic event beyond mere hypoxia-reoxygenation. Necrotic cells release endogenous alarmins, or damage-associated molecular patterns (DAMPs), including high-mobility group box 1 (HMGB1), mitochondrial DNA, ATP, and heat-shock proteins. These DAMPs engage pattern recognition receptors such as Toll-like receptors (TLR2/4) and NOD-like receptors, activating downstream cascades (Wu et al., 2024; Rao et al., 2024). HMGB1 acts as a dual-function alarmin: it binds TLR4 to activate NF-κB and drive proinflammatory cytokine (e.g., TNF-α, IL-6) transcription, and also serves as a ligand for the RAGE receptor, enhancing cell migration and inflammatory amplification. Moreover, extracellular HMGB1 promotes NLRP3 inflammasome assembly and activation, leading to caspase-1-mediated cleavage and maturation of IL-1β and IL-18 precursors (Awad et al., 2024). Thus, via the DAMPs-PRRs axis, IRI sets the initial “inflammatory tone” and establishes the cytokine/chemokine framework necessary for subsequent adaptive immunity.

Neutrophils play a pivotal role in this early phase. Once considered short-lived phagocytes, neutrophils are now recognized to exert sustained damaging effects in transplantation through neutrophil extracellular trap (NET) formation. NETosis is an active process dependent on peptidylarginine deiminase 4 (PAD4)-mediated histone citrullination (Wang Y. et al., 2024). NETs consist of chromatin DNA scaffolds decorated with myeloperoxidase, neutrophil elastase, and citrullinated histones. These structures not only physically trap pathogens but also directly damage graft vascular endothelium. Furthermore, NETs constitute a persistent reservoir of DAMPs; components such as citrullinated histone H3 can be taken up and processed by antigen-presenting cells (APCs) for presentation via MHC molecules to T cells, thereby breaking self-tolerance. This mechanism provides novel insights into *de novo* donor-specific antibodies (DSA) generation and antibody-mediated rejection (AMR) (Shu et al., 2024). Clinical studies show significantly elevated citrullinated protein levels in serum after liver reperfusion compared to pre-transplant levels [median post-reperfusion: 1.2 ng/mL vs. pre-transplant: 0.5 ng/mL, *p* < 0.0001], with higher citrullinated histone levels correlating with in-hospital mortality (OR = 1.168, *p* < 0.05) (Yokoyama et al., 2023). In animal models, PAD4 inhibition attenuates NET-mediated inflammation and liver injury after ischemia (Islam and Takeyama, 2023), supporting the therapeutic potential of NET targeting. Recent evidence has further expanded the mechanistic understanding of IRI. TRPM2, a Ca^2+^-permeable oxidative stress sensor, mediates hepatic IRI via ferroptosis; TRPM2 deficiency attenuates IR-induced liver dysfunction, inflammation, and cell death by reducing mitochondrial Ca^2+^ accumulation and suppressing ALOX12 expression, thereby limiting mitochondrial lipid peroxidation (Roggla et al., 1999). The mechanistic link between ferroptosis and NET formation has recently been extended to lung transplantation. In a mouse model of lung IRI, inhibition of the ALOX12-12-HETE pathway reduced endothelial ferroptosis and subsequently decreased NET release, thereby ameliorating primary graft dysfunction, reinforcing that targeting ferroptosis-NET crosstalk may offer a unified strategy to mitigate IRI across different transplanted organs (Cheung et al., 2001). Additionally, the SETDB1/ASK1/JNK signaling axis has been identified as another key pathway in hepatic IRI; its activation promotes hepatocyte apoptosis and inflammation, and targeting this axis confers protection against IR-induced liver damage (Gammie et al., 1999).

Simultaneously, tissue-resident macrophage populations undergo profound phenotypic and metabolic reprogramming. Single-cell transcriptomics reveals remarkable heterogeneity and plasticity of post-transplant macrophages (Li et al., 2023; Xu et al., 2023). Within the injury microenvironment, signals such as GM-CSF and IFN-γ drive macrophage polarization from an M2-like phenotype (expressing CD206, arginase-1) associated with tissue repair toward a proinflammatory M1-like state (expressing iNOS, CD86) (Du et al., 2024). Underlying this shift is metabolic rewiring: M1 macrophages switch from oxidative phosphorylation to aerobic glycolysis (the “Warburg effect”). This not only meets rapid ATP demands but also allows glycolytic intermediates to directly regulate inflammation. For example, accumulated succinate stabilizes HIF-1α to promote IL-1β expression, whereas itaconate negatively regulates inflammation by inhibiting succinate dehydrogenase and IκB (Song et al., 2025). Thus, macrophage metabolic state acts as a “conductor” of immune polarity. Targeting this metabolic switch—e.g., by inhibiting the glycolytic enzyme PKM2—may offer novel strategies for modulating the early inflammatory microenvironment.

### 2.2 Dendritic cell-mediated antigen presentation and T Cell fate determination

The initiation of adaptive immunity is governed by professional antigen-presenting cells—dendritic cells (DCs). A deeper understanding of DC subsets is essential for precise immunomodulation. Conventional DCs (cDCs) are broadly divided into cDC1 and cDC2 subsets. cDC1s (expressing XCR1, CLEC9A) excel at cross-presentation, delivering exogenous antigens via MHC class I to activate CD8^+^ cytotoxic T lymphocytes (CTLs), a key pathway in direct alloimmunity (Thim et al., 2024). cDC2s (expressing CD1c, SIRPα) primarily activate CD4^+^ T helper cells via MHC class II (Galan et al., 2025). Upon maturation, DCs undergo a “metabolic switch” from oxidative phosphorylation to aerobic glycolysis, providing biosynthetic precursors to support migration, synapse formation, and cytokine production.

#### 2.2.1 Precision regulation of co-stimulatory networks

Full T cell activation requires dual signals, with the complexity of co-stimulatory pathways extending far beyond the classic CD28-B7 axis. A representative example is the TIGIT/CD226/CD155 axis. TIGIT is an inhibitory receptor containing an immunoreceptor tyrosine-based inhibitory motif (ITIM) that recruits SHP phosphatases to attenuate TCR signaling. Moreover, TIGIT competitively binds the shared ligand CD155 (PVR) against the activating receptor CD226, indirectly suppressing T cell activation at the ligand level (Shang et al., 2024). In the setting of chronic antigen exposure post-transplantation, sustained high TIGIT expression is associated with T cell exhaustion—a double-edged state that dampens anti-graft responses but also compromises anti-viral and anti-tumor immunity. Therefore, targeting this pathway requires refined strategies. For instance, engineered TIGIT-Fc fusion proteins that selectively block TIGIT-CD155 interaction while sparing CD226 activation have shown strong potential to induce antigen-specific tolerance in non-human primate models (Cai et al., 2023a). Additionally, the ICOS-ICOSL pathway plays key roles in regulatory T cell (Treg) stability and follicular helper T cell (Tfh) differentiation, representing an important target for humoral immunity regulation (Yang et al., 2021).

#### 2.2.2 Metabolic reprogramming and T Cell differentiation

The principle “signals drive metabolism, metabolism dictates fate” underpins T cell biology. Upon activation through TCR and co-stimulation, naïve T cells undergo dramatic metabolic rewiring to meet bioenergetic and biosynthetic demands for proliferation and differentiation. Effector T cells (Teff), such as Th1 and Th17, exhibit a canonical “anabolic” profile: they markedly increase glucose uptake and aerobic glycolysis, and enhance glutaminolysis to replenish TCA cycle intermediates (anaplerosis). In contrast, memory T cells (Tm) and Tregs rely more on fatty acid oxidation (FAO) and mitochondrial oxidative phosphorylation for energy production—a “catabolic” signature that supports long-term survival and functional homeostasis. Mitochondrial dynamics underlie this metabolic divergence: Teff cells display fragmented, immature mitochondria optimized for biosynthetic precursor supply, whereas Tm and Treg cells possess fused, networked mitochondria with higher membrane potential and oxidative capacity. This dynamic process is regulated by Drp1 (promoting fission) and OPA1 (promoting fusion) (Che et al., 2022). Pharmacologic inhibition of Drp1 (e.g., with Mdivi-1) preferentially promotes memory-like T cell generation (Tran et al., 2022), offering an exciting strategy to induce protective rather than destructive immune memory post-transplantation.

#### 2.2.3 Establishment of epigenetic memory

If metabolic reprogramming determines T cells’ “immediate fuel,” epigenetic modifications engrave their “long-term identity code.” Transcriptional programs during T cell differentiation are stabilized and maintained through histone modifications, DNA methylation, and chromatin accessibility. In Teffs, loci associated with effector functions (e.g., IFN-γ, IL17) are enriched with active marks such as H3K4me3 and H3K27ac at promoters and enhancers, while genes related to regulatory functions (e.g., FOXP3, IL2RA) may be silenced by repressive marks like H3K27me3. Tissue-resident memory T cells (TRM), which constitute a major tolerance barrier within the graft, undergo even more profound epigenetic reprogramming. Genome-wide chromatin accessibility analysis (ATAC-seq) shows that TRM cells maintain open chromatin at loci encoding tissue-residency molecules (CD69, CD103, ITGAE) and effector molecules (Granzyme B), while loci for lymph-node homing receptors (CCR7, S1PR1) are closed (Li et al., 2025). Emerging “epigenetic editing” technologies, which fuse catalytically dead dCas9 to epigenetic modifiers (e.g., DNMT3A for silencing or TET1 for activation), enable precise rewriting of epigenetic marks at specific genomic loci (Capelletti et al., 2024). For instance, targeted methylation of the IFN-γ or IL17 promoter regions has successfully suppressed Th1 or Th17 differentiation in experimental models (Liu R. et al., 2023), demonstrating unprecedented potential for achieving durable, potentially heritable immune tolerance. To provide a concise overview of the key intervention strategies discussed in this section, Table 1 summarizes representative approaches, their mechanisms, and current developmental stages, primarily based on studies published within the last two to 3 years.

**TABLE 1:** Summary of representative precision immunomodulation strategies.

Strategy category	Representative agent/Technology	Mechanism of action	Current development stage and evidence level
Engineered cellular therapy	HLA-A2 CAR-Treg	Antigen-specific homing and local suppression	Preclinical (mouse heart transplant model)
Engineered cellular therapy	CRISPR-dCas9-TET1 (FOXP3 CNS2 demethylation)	Epigenetic stabilization of Treg lineage	Preclinical proof-of-concept
Targeted biologics	Iptacopan (complement factor B inhibitor)	Alternative pathway inhibition in AMR	Clinical phase II (recurrent IgA nephropathy post-kidney transplant, n = 5)
Targeted biologics	Daratumumab (anti-CD38)	Plasma cell depletion for refractory AMR	Clinical case series
Local microenvironment remodeling	PLGA-CTLA4-Ig implant	Sustained local tolerance niche around the graft	Preclinical (mouse)
Local microenvironment remodeling	pH/ROS-responsive nanocarriers	Stimuli-triggered drug release at rejection site	Preclinical
Metabolic intervention	Mdivi-1 (Drp1 inhibitor)	Promotes memory-like T cell generation	Preclinical
Epigenetic intervention	dCas9-DNMT3A (IFN-γ promoter methylation)	Permanent silencing of effector T cell function	Preclinical

### 2.3 Mechanisms of long-term immunological memory barriers

#### 2.3.1 Characteristics and functions of tissue-resident memory T Cells

TRMs constitute the most persistent immunological memory barrier within the graft, acting as resident “sentinels” that mount rapid responses to local antigen re-encounter. Their formation and maintenance involve multi-step processes regulated by numerous factors. Initially, circulating precursor T cells are “intercepted” in tissues through combined antigenic and microenvironmental signals, particularly TGF-β and IL-15. TGF-β induces expression of the integrin CD103 (αEβ7), which binds E-cadherin on epithelial cells to anchor TRM within the tissue. IL-15, via trans-presentation, provides essential survival signals (Kurd et al., 2020). Single-cell sequencing has revealed significant functional heterogeneity within TRM populations, encompassing terminally differentiated effector TRM (expressing GZMB, IFN-γ) and a precursor-like subset with self-renewal capacity and stem-cell-associated gene expression (TCF7, IL7R) (Hee et al., 2023). The latter likely serves as a reservoir for TRM pool maintenance. Emerging research highlights TRM’s unique metabolic adaptability: compared to circulating memory T cells, TRM exhibit enhanced fatty acid oxidation and mitochondrial fitness, possibly reflecting adaptation to local nutrient availability (Wishart et al., 2025). Notably, TRM metabolic profiles are tissue-specific. Kidney TRM, residing in a relatively hypoxic microenvironment, displays dual dependency on fatty acid oxidation and lactate utilization mediated by the lactate transporter MCT1, a key survival mechanism in inflammatory and hypoxic settings (Singh et al., 2023). Such tissue-specific metabolic programs represent core adaptations to distinct tissue microenvironments (e.g., skin vs. gut) (Rei et al., 2021). Functional studies confirm that targeting key steps in fatty acid oxidation disrupts TRM survival and function, validating the potential of metabolism-based interventions (Chen et al., 2023).

#### 2.3.2 Complexity of humoral immune responses

Humoral immunity, particularly DSA production, is a central mechanism driving chronic graft dysfunction. Its complexity spans multiple levels: generation pathways, cellular maintenance, and antibody functionality.

First, DSA can originate via “fast and slow” pathways. The traditional view emphasizes DSA generation through germinal center (GC) B cells undergoing somatic hypermutation and affinity maturation. However, the rediscovery of “extrafollicular plasma cell responses” reveals an alternative, rapid, and efficient pathway (Xu J. et al., 2025). Under strong inflammatory signals (e.g., IFN-γ, IL-21), activated B cells can differentiate directly into short-lived plasma cells at the T-B cell border, rapidly producing large quantities of (often lower-affinity) antibodies, thereby driving early AMR (Louis et al., 2022).

Second, long-lived plasma cells sustain persistent DSA production. These cells primarily reside in specialized survival niches such as the bone marrow. The CXCL12 (secreted by stromal cells)-CXCR4 (expressed on plasma cells) axis recruits and anchors plasma cells within the niche, while the APRIL-BCMA axis delivers critical survival signals that prevent apoptosis (Aradottir Pind et al., 2022; Lee and Linterman, 2022). The distribution and properties of plasma cell niches vary across transplant types (e.g., bone marrow, the graft itself, mucosal lymphoid tissues), explaining why certain transplants (e.g., intestinal) are more prone to refractory AMR (Heeger et al., 2024).

**FIGURE 1:**
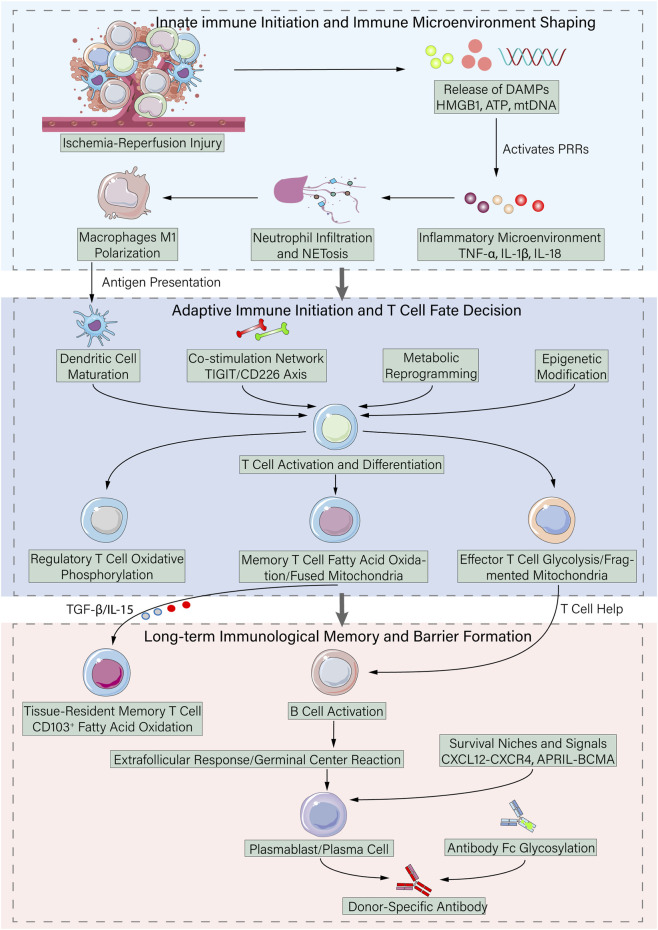
Hierarchical network of the alloimmune response in transplantation. This schematic summarizes key rejection mechanisms, progressing from innate immune activation to adaptive immunity and long-term memory formation. Initiated by ischemia-reperfusion injury (IRI) and DAMPs, the process triggers NETosis and macrophage M1 polarization, shaping the inflammatory microenvironment. Dendritic cells then activate T cells, whose fate is determined by co-stimulation, metabolic reprogramming, and epigenetic modification, leading to differentiation into effector, memory, or regulatory subsets. Finally, tissue-resident memory T cells (TRM) and donor-specific antibodies (DSA) produced by plasma cells form persistent memory barriers that sustain graft rejection. This framework highlights potential targets for precise immunomodulation.

Finally, antibody “quality” determines pathogenicity. DSA titer alone is insufficient to predict graft injury risk. The Fab region mediates antigen recognition, with affinity being a critical factor. The Fc region directly mediates tissue injury: IgG Fc can activate complement via the classical pathway or engage FcγR on NK cells and macrophages to mediate antibody-dependent cellular cytotoxicity (ADCC). Recent studies highlight Fc glycosylation as a “molecular switch” regulating these effector functions: absence of core fucosylation significantly enhances binding to FcγRIIIa, boosting ADCC activity (Bottinger et al., 2024), whereas increased terminal galactosylation promotes C1q binding and complement-dependent cytotoxicity (CDC) (Cvijic et al., 2024). Thus, future clinical monitoring must evolve from assessing “quantity” to evaluating “quality,” incorporating surface plasmon resonance (SPR) for affinity analysis and mass spectrometry for DSA glycan profiling to better assess pathogenic risk and guide therapy (Figure 1).

## 3 Innovations and breakthroughs in Frontier intervention strategies

Deepened understanding of the alloimmune network has catalyzed a therapeutic revolution from “broad suppression” to “precision reconstruction.” Current trends are shifting from global immunosuppression toward precise modulation of specific cell subsets, signaling pathways, and local microenvironments. This section outlines four pillar strategies—engineered cellular therapies, novel biologics, local microenvironment intervention, and metabolic/epigenetic modulation—that collectively constitute the toolbox for the era of precision transplant immunology.

### 3.1 A new era of cell therapy: actively establishing tolerance

To overcome long-term memory barriers formed by TRM and long-lived plasma cells (Section 2.3), cell-based strategies aim to “educate” or engineer patient-derived immune cells with specific regulatory functions for reinfusion, actively establishing tolerance and shifting the paradigm from “drug maintenance” to “cellular cure.”

#### 3.1.1 Engineered tregs

Treg therapy is a cornerstone strategy for transplant tolerance but faces challenges regarding stability, specificity, and function. Next-generation engineering approaches are systematically addressing these issues. To enhance specificity, chimeric antigen receptor (CAR) technology enables Tregs to precisely recognize graft antigens. For example, HLA-A2-targeted CAR-Tregs specifically home to and accumulate in grafts expressing this antigen, exerting localized suppression. Preclinical data in a mouse heart transplant model show that HLA-A2 CAR-Treg treatment (n = 4) achieved a median graft survival of 99 days, significantly outperforming polyclonal Tregs (35 days, n = 4) and controls (23 days, n = 7). Histopathological analysis confirmed reduced lymphocyte infiltration in the CAR-Treg group (Wagner et al., 2022). To improve stability, epigenetic engineering provides tools to “lock in” the Treg lineage. The epigenetic state of the FOXP3 locus, particularly demethylation of its conserved non-coding sequence 2 (CNS2) region, is crucial for Treg stability under inflammatory conditions. Using low-dose DNA methyltransferase inhibitors (e.g., decitabine) or targeted delivery of a CRISPR-dCas9-TET1 demethylation system to CNS2 can actively maintain FOXP3 expression, preventing conversion to ex-Tregs that secrete IFN-γ (Murakam et al., 2023). To augment functionality, metabolic engineering enhances Treg fitness in the stressful graft microenvironment. Treg suppressive function relies heavily on mitochondrial oxidative phosphorylation. Overexpression of PGC1α, a master regulator of mitochondrial biogenesis, improves mitochondrial quality, respiratory capacity, and ATP production, thereby boosting Treg survival and function in competitive inflammatory settings (Fang et al., 2023). In summary, CARs provide “navigation,” epigenetic engineering ensures “loyalty,” and metabolic engineering furnishes “endurance,” rendering next-generation engineered Tregs increasingly intelligent and potent.

#### 3.1.2 Other cell therapy strategies

Regulatory dendritic cells (DCregs) function as “tolerogenic sentinels.” Their core mechanism involves “incomplete activation”: DCregs loaded with donor antigen are infused in an immature or tolerogenic state, characterized by low co-stimulatory molecule (CD80/86) expression and high secretion of immunomodulatory cytokines (IL-10, TGF-β), thereby inducing T cell anergy or promoting Treg generation. Emerging techniques use nanoparticles to deliver antigens and regulatory molecules, enabling more precise programming of DCregs *ex vivo*. Co-encapsulation of donor MHC peptides with rapamycin or vitamin D3 analogs into PLGA nanoparticles allows sustained intracellular release upon DC uptake, inducing DCregs with more stable phenotypes and superior tolerogenic efficacy in animal models compared to conventionally generated DCregs (Kammona and Kiparissides, 2020; Luo et al., 2017).

Mesenchymal stromal cells (MSCs) act as “mobile pharmacies” and “microenvironment architects.” Their immunomodulatory effects are largely mediated through paracrine mechanisms, with extracellular vesicles—particularly exosomes—identified as key effectors. MSC-derived exosomes carry abundant immunomodulatory molecules (e.g., TGF-β, GAL-9, miRNA-let7c) that inhibit T cell proliferation, promote macrophage M2 polarization, and increase Treg proportions (Hua et al., 2025). Compared to MSCs, exosomes exhibit lower tumorigenic and immunogenic risks, alongside easier storage and standardization, positioning them as rising stars in cell therapy (Zhao et al., 2025). Additionally, MSCs can directly donate functional mitochondria to damaged tissue cells (e.g., renal tubular epithelial cells) via mitochondrial transfer, enhancing tissue repair and indirectly suppressing inflammation (Jaraba-Alvarez et al., 2025).

### 3.2 Precision targeting with novel biologics

Novel biologics aim to precisely block key immune pathways while preserving overall immune defense.

#### 3.2.1 Specific blockade of co-stimulatory pathways

Targeted co-stimulation blockade has entered a “second generation,” marked by greater potency and improved safety. The CD40^−^CD40L pathway exemplifies this evolution. First-generation anti-CD40L antibodies failed due to Fc receptor-mediated platelet aggregation and thrombosis. Newer antibodies (e.g., ASKP1240) employ engineered IgG4 backbones or point mutations that eliminate Fc receptor binding, successfully averting thrombotic complications while maintaining potent immunosuppressive efficacy and prolonging graft survival in non-human primate models (Rech Tondin and Lanzoni, 2025). Another trend is combination blockade, given the redundancy in immune activation signals. Simultaneous blockade of CD28 and CD40L pathways produces strong synergy in primate models, inducing long-term transplant tolerance, whereas blocking either pathway alone shows limited efficacy (Del Bello and Treiner, 2023). Mechanistically, CD28 provides initial T cell activation and survival signals, while CD40L is essential for DC “licensing”; dual blockade thus disrupts both upstream and downstream events of T cell activation, achieving more comprehensive immune quiescence.

#### 3.2.2 Multi-level control of antibody-mediated rejection

Managing AMR requires multi-level intervention targeting antibody generation, maintenance, and effector functions. At the effector level, complement inhibition has expanded beyond terminal pathway C5 inhibitors (e.g., eculizumab) to upstream targets. Inhibitors of complement factor B (e.g., iptacopan) specifically dampen the alternative pathway, often hyperactivated in AMR, while better preserving classical pathway immune surveillance, thereby mitigating infection risks (Troise et al., 2025). In a clinical trial involving kidney transplant recipients with recurrent IgA nephropathy (n = 5, biopsy-confirmed), iptacopan treatment for 6–12 months significantly reduced terminal complement component (C5b-9) deposition, nearly eliminated C3, and slightly reduced IgA deposition, with no major adverse events reported (Sanna et al., 2025). These findings support larger cohort studies to define iptacopan’s impact on long-term clinical outcomes. For plasma cell depletion, antibodies targeting CD38 (highly expressed on plasma cells), such as daratumumab, show considerable promise. Next-generation anti-CD38 antibodies feature Fc-engineered enhancements that bolster ADCC and CDC, more effectively eliminating both short- and long-lived plasma cells and offering a new option for refractory AMR (Randone et al., 2024). To disrupt plasma cell survival niches, CXCR4 antagonists (e.g., plerixafor) can mobilize bone marrow plasma cells into the periphery, depriving them of niche protection and sensitizing them to chemotherapy or monoclonal antibody therapy, thereby providing a novel strategy to eradicate DSA sources (Su et al., 2021).

### 3.3 Precision remodeling of the local microenvironment

The essence of local microenvironment remodeling is to “deliver the right drug to the right place at the right time and at the right dose,” maximizing efficacy while minimizing systemic toxicity.

Smart nanocarriers serve as an ideal platform. Surface functionalization enables active targeting—for instance, conjugating ligands for VCAM-1 or E-selectin directs nanocarriers to activated vascular endothelium associated with AMR, achieving vessel-specific drug delivery (Cong et al., 2024). Stimuli-responsive carriers enable intelligent drug release triggered by pathological microenvironment features: pH-sensitive nanoparticles undergo structural changes in the acidic environment of inflamed areas (pH ∼6.5–6.8) for rapid drug release (Anbazhagan and Mahalingam, 2025); redox-sensitive carriers exploit high reactive oxygen species (ROS) levels at rejection sites, where cleavage of disulfide bonds triggers localized “burst” release (Shi W. et al., 2025).

Biomaterial scaffolds aim to construct a durable “immune-tolerant niche” at the transplant site. For example, embedding CTLA4-Ig within biodegradable poly (lactic-co-glycolic acid) (PLGA) microspheres or hydrogels placed under the renal capsule or around islet grafts enables sustained local release over weeks to months. This creates a high-concentration “tolerance zone” around the graft that not only suppresses local effector T cells but also actively recruits and expands host-derived Tregs, fostering a self-sustaining local tolerant milieu (Bentley and Little, 2021). Efficacy and safety assessments in mouse models show that renal subcapsular PLGA-CTLA4-Ig implants significantly prolong allograft survival, reduce inflammatory infiltrates, and exhibit good biocompatibility without impairing graft function (Kuppan et al., 2023). Even more promising are inherently immunomodulatory biomaterials: hydrogels constructed from succinate-modified hyaluronic acid degrade to release succinate, which itself promotes macrophage M2 polarization via HIF-1α stabilization, thereby actively shaping an anti-inflammatory, pro-repair microenvironment while supporting tissue regeneration (Xu X. et al., 2025).

### 3.4 Novel strategies for metabolic and epigenetic intervention

#### 3.4.1 Targeting metabolic checkpoints

The core rationale of metabolic intervention is to exploit “preferential differences” in metabolic programs among immune cell subsets for selective modulation. mTOR inhibitors (e.g., sirolimus) are pioneers in this area, differentially affecting T cell subset metabolism: effector T cell activation and function heavily depend on mTORC1-driven glycolysis, whereas Treg differentiation and function rely more on mTORC2 signaling and fatty acid oxidation. Thus, appropriate dosing of mTOR inhibitors can suppress Teff while relatively sparing or even promoting Treg function (Tai et al., 2025). Intermittent dosing may enhance this selectivity, possibly because Tregs recover faster from metabolic stress than Teffs. Targeting amino acid metabolism also holds great promise. Indoleamine 2,3-dioxygenase (IDO), a key enzyme in tryptophan metabolism expressed by DCs or stromal cells, converts tryptophan to kynurenines that activate the aryl hydrocarbon receptor (AhR) and the amino acid response (GCN2 kinase), thereby inhibiting T cell function and promoting Tregs (Tao et al., 2025). Glutamine antagonists (e.g., JHU083) impede pyrimidine and purine synthesis in T cells, effectively curbing clonal expansion and showing robust efficacy in autoimmunity and transplant models (Shi Y. et al., 2025).

#### 3.4.2 Therapeutic potential of epigenetic modulation

Epigenetic therapy aims to reprogram the “hardware” of immune cells for durable, even permanent, phenotypic change. Histone deacetylase (HDAC) inhibitors increase histone acetylation, relaxing chromatin structure and altering gene expression profiles. Notably, different HDAC isoforms play opposing roles in T cell differentiation: inhibiting HDAC6 promotes FOXP3 expression and Treg function, whereas inhibiting HDAC9 enhances Teff responses (Zhang et al., 2023; von Knethen et al., 2020). This isoform specificity guides the development of precise epigenetic drugs. DNA methyltransferase (DNMT) inhibitors (e.g., decitabine) induce DNA hypomethylation, potentially “reactivating” genes silenced by hypermethylation, such as regulatory pathways in Teffs (Crossland et al., 2020). However, their lack of specificity remains a major challenge. Epigenetic editing represents the ultimate in precision: using dCas9-DNMT3A or dCas9-TET1 systems allows precise methylation or demethylation at specific loci—for instance, demethylating the FOXP3 CNS2 region to stabilize its expression (Xu et al., 2016) or hypermethylating the IFN-γ promoter to silence it (Liu et al., 2024). Such site-specific “epigenetic surgery” offers a powerful tool for achieving durable, antigen-specific immune tolerance.

## 4 Establishing and implementing the precision medicine paradigm

Success in transplantation immunology depends not only on advanced therapeutics but also on precise, dynamic insight into individual immune status. The precision medicine paradigm integrates multidimensional data to construct a digital immune profile for each patient, transforming treatment decisions from “population-based experience” to “individualized customization.”

### 4.1 Development and application of novel biomarkers

Donor-derived cell-free DNA (dd-cfDNA) has emerged as a non-invasive “liquid biopsy” tool for monitoring graft injury. Its sensitivity and specificity exceed 90%, enabling earlier rejection detection than conventional creatinine monitoring (Liu et al., 2024). Advanced fragmentomics analysis further distinguishes injury mechanisms by deep sequencing of dd-cfDNA fragment size, end motifs, and nucleosome positioning. For instance, dd-cfDNA fragmentation patterns released during ischemia-reperfusion injury differ from those associated with T cell-mediated rejection (enriched in short fragments from actively transcribed genes) or antibody-mediated rejection (specific end motifs) (Akifova et al., 2024). This “non-invasive phenotyping” capability is critical for precise clinical intervention.

DSA monitoring has entered a “functional era.” SPR measures real-time antibody-antigen binding kinetics (Kon, Koff, KD) without labeling, providing more accurate affinity data than ELISA (Wang S. et al., 2024). Functional cell-based assays—such as C1q/C3d binding assays and more sensitive endothelial cell activation tests—directly evaluate DSA capacity to activate complement and provoke inflammation (Cheng et al., 2025; Zhou et al., 2024). Combined with Fc glycan profiling, a comprehensive DSA risk assessment system is emerging to better identify “high-risk” DSAs and guide preemptive therapy.

### 4.2 Advances in high-dimensional immune profiling

Single-cell multi-omics technologies (e.g., CITE-seq, ATAC-seq) enable unprecedented resolution of the post-transplant immune landscape. By simultaneously capturing the transcriptome, hundreds of surface proteins, and T cell receptor (TCR) sequences from single cells, these tools map continuous differentiation trajectories and track clonal expansion and evolution of specific T cell populations during rejection or tolerance (Trzupek et al., 2020). During acute rejection, T cell clones co-expressing tissue-residency and cytotoxic genes emerge within the graft and can also be detected peripherally, serving as early warning biomarkers (Palia et al., 2023).

Spatial transcriptomics/proteomics platforms (e.g., GeoMx DSP, CosMx, MIBI-TOF) restore biological context, revealing the precise anatomical localization of activated T cells (e.g., within renal tubulointerstitium vs. glomeruli), their interactions with nearby APCs or stromal cells, and associated signaling pathways (Giarraputo et al., 2025). In chronic graft dysfunction, these technologies have identified unique macrophage-fibroblast niches surrounding fibrotic areas, offering specific targets to interrupt pathological tissue remodeling.

### 4.3 Integration of artificial intelligence and predictive modeling

Machine learning and deep learning are becoming central engines for processing high-dimensional, multimodal transplant data. Integrating electronic health records, laboratory results, dd-cfDNA levels, and single-cell immune profiles enables models that individually predict rejection risk. Recurrent neural networks (RNNs) and long short-term memory networks (LSTMs) are particularly suited for time-series data, capturing dynamic immune evolution for true early warning (Yi et al., 2024). In a single-center validation study involving 1893 subjects (591 liver transplant recipients with at least one biopsy and 1302 controls), a weighted LSTM model predicted liver fibrosis with an AUC of 0.798 (95% CI: 0.790–0.810), outperforming unweighted LSTM (0.761), RNN (0.736), temporal convolutional network (0.700), and random forest (0.679) (Azhie et al., 2023), demonstrating strong generalizability.

The digital twin concept elevates individualized modeling by creating a virtual “digital copy” of each patient, integrating physiologically based pharmacokinetic (PBPK), pharmacodynamic (PD), and immunodynamic parameters. Clinicians can simulate different immunosuppressive regimens (e.g., tacrolimus target concentration adjustments, combination therapies) on the digital twin to predict efficacy and side effects before real-world intervention, optimizing decision-making (Iyer and Umadevi, 2025; Baumgartner and Brislinger, 2025). In a clinical study, Halder et al. (2025) developed PePMDT, a digital twin model combining deep learning with mathematical modeling to predict donor-specific gene expression dynamics during liver regeneration post-transplantation. Using blood samples from 12 donors and serial post-operative gene expression measurements, the model accurately reconstructed future expression profiles, demonstrating high predictive accuracy and clinical utility. Further advances integrate organ-on-a-chip systems with computational models, constructing simplified “graft models” from patient cells on microfluidic chips to test biologic efficacy and improve in vitro–in vivo correlation (Geng et al., 2024).

### 4.4 Optimization of individualized dosing strategies

PBPK model-guided dosing is advancing beyond traditional therapeutic drug monitoring (TDM). These models incorporate patient age, weight, liver/kidney function, CYP3A5/ABCB1 genotypes, and concomitant medications to better predict initial doses and exposure of drugs like tacrolimus, significantly shortening time to target therapeutic range (Cai et al., 2023b).

Incorporating pharmacodynamic (PD) biomarkers aims to directly read the immune system’s response to therapy, shifting focus from “pharmacokinetics (what the body does to the drug)” to “pharmacodynamics (what the drug does to the body).” For example, measuring NFAT-regulated gene expression (NRGE) quantifies tacrolimus-mediated inhibition of the TCR-NFAT pathway, while flow cytometric detection of pS6 (downstream of mTOR) assesses sirolimus activity (38799459). PD-guided dosing may better reflect a patient’s true immunosuppressive state than TDM alone, potentially optimizing the balance between rejection prevention and excessive immunosuppression.

## 5 Future perspectives and challenges

Despite transformative progress, transplant immunology stands at the threshold of a new era. Future breakthroughs will require solving remaining scientific puzzles and thoughtfully integrating cutting-edge technologies.

### 5.1 Breakthroughs and hurdles in xenotransplantation

Xenotransplantation offers a fundamental solution to the organ shortage. CRISPR-Cas9 multi-gene editing now enables simultaneous knockout of three major carbohydrate antigen genes in pigs (GGTA1, CMAH, β4GalNT2) and insertion of multiple human transgenes encoding complement regulators (hCD46, hCD55, hCD59) and thrombomodulin (hTBM). Organs from these “10-gene edited pigs” have achieved survival times of months to over a year in non-human primates, marking a historic breakthrough (Singh et al., 2025; Singh et al., 2022). Nevertheless, interspecies incompatibility remains a major hurdle: thrombotic microangiopathy persists as a key obstacle, driven by dysregulated interactions between porcine endothelial cells and human coagulation systems. Mechanistic studies highlight the role of high tissue factor (TF) expression on porcine endothelium, which aberrantly activates human factor VII (FVII) and triggers uncontrolled coagulation (Iwase et al., 2014). Targeting this mechanism with siRNA nanoparticles against porcine TF effectively reduces TF expression, attenuates thrombosis, and prolongs xenograft survival in preclinical models (Reichart et al., 2023). Another barrier involves the SIRPα-CD47 “don’t eat me” signaling axis: weak interaction between porcine CD47 and human SIRPα fails to inhibit human macrophage phagocytosis, whereas transgenic expression of human CD47 significantly suppresses this response (Nomura et al., 2020). Regarding safety, CRISPR-Cas9-mediated complete eradication of porcine endogenous retrovirus (PERV) copies from the pig genome prevents cross-species viral transmission, addressing a critical long-term safety concern (Niu et al., 2017).

### 5.2 Application prospects of spatial multi-omics

Spatial multi-omics will serve as the next decade’s “microscope” for understanding transplant immunology complexity. Future directions include achieving subcellular resolution and integrating full molecular dimensions (transcriptome, proteome, metabolome, epigenome). High-resolution spatial transcriptomics could reveal stable immune synapses between specific T cell clones and APCs at sites of chronic vascular rejection and identify critical ligand-receptor pairs (e.g., CD40^−^CD40L, LFA-1-ICAM-1) (Schafer et al., 2024; Zh et al., 2023), providing a precise blueprint for ultra-localized interventions such as blocking nanobodies.

### 5.3 Immune aging and personalized therapy

Immunosenescence presents unique challenges for transplant recipients, especially the growing elderly population. The aged immune system is characterized by a contracted TCR repertoire, depleted naïve T cell pools, accumulated memory/effector T cells, and chronic low-grade inflammation (“inflammaging”). Single-cell studies reveal distinct epigenetic aging signatures (e.g., hypermethylation at specific loci) and metabolic inertia (mitochondrial dysfunction) in aged T cells (Liu Z. et al., 2023; Sun et al., 2023). This demands tailored immunosuppressive regimens for elderly recipients—potentially lower-intensity but more targeted against inflammaging itself. Novel biomarkers like “epigenetic clocks,” which quantify biological age and immunosenescence via CpG methylation analysis, may offer more reliable guidance for personalized dosing than chronological age alone (Tomusiak et al., 2024).

Clinical translation studies are exploring biomarker-guided individualized immunosuppression. For instance, assessing recipient immunosenescence via epigenetic clocks has been proposed to adjust immunosuppressive intensity dynamically, aiming to better balance rejection control against infection risk from over-immunosuppression, particularly in older recipients (Horvath and Raj, 2018). Concurrently, novel interventions targeting TRM—a persistent therapeutic hurdle—focus on combined disruption of their survival-dependent metabolic and epigenetic mechanisms (Prosser et al., 2018). Preclinical proof-of-concept suggests this dual approach may clear TRM more effectively than single therapies while better preserving systemic immune memory, offering a promising direction for future precision immunomodulation.

### 5.4 Technological innovation and clinical translation

Translating scientific discoveries into routine clinical practice requires addressing standardization, automation, and regulatory challenges. Closed automated cell manufacturing systems are key to ensuring quality, consistency, and accessibility of advanced therapies like CAR-Tregs. Off-target effects and long-term safety of gene-editing technologies necessitate unified evaluation standards and regulatory frameworks.

Innovative clinical trial designs—such as adaptive, umbrella, and basket trials—allow protocol adjustments based on interim analyses or testing of multiple therapies within biomarker-defined subgroups under a single framework, greatly enhancing drug development efficiency (James et al., 2024; Doenst et al., 2025). Systematic collection and analysis of real-world evidence (RWE) will provide indispensable complementary data on long-term benefit-risk profiles of new therapies in broader populations.

### 5.5 Ethical and long-term safety considerations

While the technological breakthroughs discussed above—such as gene editing, xenotransplantation, and AI-driven digital twins—hold immense promise, they also raise important ethical and safety questions that require parallel attention. For xenotransplantation, beyond the well-recognized risk of porcine endogenous retrovirus transmission, the welfare of genetically engineered pigs and the informed consent process for recipients deserve clearer regulatory frameworks. For gene editing applied to cellular therapies (e.g., CAR-Tregs or epigenetic editing), the potential for off-target mutagenesis and clonal expansion remains a long-term safety concern, especially when aiming for permanent epigenetic rewriting. Moreover, digital twins and AI-based predictive models rely on large-scale patient data, which introduces risks related to data privacy, algorithmic bias, and model generalizability across diverse populations. These challenges are not insurmountable, but they must be proactively addressed through interdisciplinary collaboration among immunologists, bioethicists, regulators, and patient advocates. Future clinical translation will depend as much on robust ethical oversight as on scientific efficacy.

### 5.6 Discovery and validation of novel targets

Systems biology approaches will continue to drive novel target discovery. Integrating large-scale single-cell datasets to construct gene co-expression and regulatory networks can identify core regulatory nodes driving rejection or tolerance. Comparative immune profiling of tolerant versus rejecting patients has already revealed gene modules associated with stable tolerance (e.g., Treg modules expressing CTLA4, LAG3, IL10) (Lopez-Lopez et al., 2021).

The non-coding RNA world (microRNA, lncRNA, circRNA) represents a vast regulatory repository. Certain lncRNAs highly expressed in grafts (e.g., LNCRNA-ACOD1) may influence the local immune microenvironment by regulating metabolic enzyme expression, offering organ-specific targets (Nog et al., 2022). Furthermore, deep investigation of post-translational modification (PTM) networks (phosphorylation, ubiquitination, lactylation, etc.) will uncover rapid, dynamic regulatory layers in immune signaling, often ideal for small-molecule inhibitor development and expanding the precision therapeutic arsenal.

### 5.7 A developmental roadmap for the next 5–10 years

Looking ahead, several specific breakthroughs are poised for near-term clinical translation. First, CAR-Treg therapies for solid organ transplantation are expected to enter early-phase clinical trials within the next three to 5 years, driven by advances in closed automated manufacturing and safety switches. Second, local delivery systems using smart nanocarriers or biomaterial scaffolds will likely be the first to combine with existing immunosuppressants, offering reduced systemic toxicity without requiring complete drug replacement. Third, complement inhibitors targeting the alternative pathway (e.g., iptacopan) are already in clinical testing for recurrent glomerular diseases post-transplant and may be repurposed for antibody-mediated rejection within 5 years. Fourth, epigenetic editing remains at the preclinical stage, but the next decade could see *ex vivo* applications—such as stabilizing FOXP3 expression in Tregs before infusion—as a practical intermediate step. Finally, spatial multi-omics and artificial intelligence will shift from discovery tools to clinical adjuncts, enabling biopsy-free rejection phenotyping and individualized risk forecasting. The challenge is not a lack of innovation but prioritization and integration. We propose that the most impactful near-term goal is not to achieve complete immune tolerance in all patients, but to convert long-term graft survival from a 10-year horizon to a 20-year or lifelong reality through layered precision modulation.

## 6 Conclusion

In summary, transplantation immunology is undergoing a profound paradigm shift driven by basic research and empowered by technological innovation. The core transition is moving from non-specific, systemic immunosuppression toward precision remodeling of the local immune microenvironment and exact modulation of specific immune cell functions. Engineered cellular therapies, targeted biologics, smart delivery systems, and metabolic/epigenetic interventions collectively constitute an increasingly powerful toolbox for precision immunomodulation.

Looking forward, the comprehensive establishment of a precision medicine paradigm remains the ultimate goal. Integrating multi-omics monitoring, AI-powered predictive modeling, and individualized dosing systems promises to create a unique “immune digital twin” for each transplant recipient, enabling precision management from early rejection risk warning to dynamic treatment optimization. Although challenges persist—including complete eradication of TRM, overcoming xenotransplantation barriers, optimizing individualized regimens, and translating innovative technologies to the clinic—interdisciplinary collaboration is steadily advancing the field toward a new era. This era holds the promise of achieving durable immune coexistence without long-term potent immunosuppression, ultimately offering transplant recipients longer graft survival and higher quality of life.

## References

[B1] Akifova A. Budde K. Oellerich M. Beck J. Bornemann-Kolatzki K. Schutz E. (2024). Perspective for donor-derived cell-free DNA in antibody-mediated rejection after kidney transplantation: defining context of use and clinical implications. Transpl. Int. 37, 13239. 10.3389/ti.2024.13239 39188271 PMC11345135

[B2] Anbazhagan M. K. Mahalingam S. (2025). Advancements in nanofibers and nanocomposites: cutting-Edge innovations for tissue engineering and drug delivery-A review. Sci. Prog. 108 (2), 368504241300842. 10.1177/00368504241300842 40375476 PMC12081990

[B3] Aradottir Pind A. A. Thorsdottir S. Magnusdottir G. J. Meinke A. Del Giudice G. Jonsdottir I. (2022). A comparative study of adjuvants effects on neonatal plasma cell survival niche in bone marrow and persistence of humoral immune responses. Front. Immunol. 13, 904415. 10.3389/fimmu.2022.904415 35990686 PMC9381929

[B4] Awad A. M. Elshaer S. L. Gangaraju R. Abdelaziz R. R. Nader M. A. (2024). Ameliorative effect of montelukast against STZ induced diabetic nephropathy: targeting HMGB1, TLR4, NF-kappaB, NLRP3 inflammasome, and autophagy pathways. Inflammopharmacology 32 (1), 495–508. 10.1007/s10787-023-01301-1 37498374 PMC10907471

[B5] Azhie A. Sharma D. Sheth P. Qazi-Arisar F. A. Zaya R. Naghibzadeh M. (2023). A deep learning framework for personalised dynamic diagnosis of graft fibrosis after liver transplantation: a retrospective, single Canadian centre, longitudinal study. Lancet Digit. Health 5 (7), e458–e466. 10.1016/S2589-7500(23)00068-7 37210229

[B6] Bao J. Wen Y. Tian L. Jie Y. (2025). Decoding allergic conjunctivitis: latest perspectives on etiological drivers and immunopathological mechanisms. Clin. Rev. Allergy Immunol. 68 (1), 85. 10.1007/s12016-025-09098-3 40879847 PMC12397188

[B7] Baumgartner C. Brislinger D. (2025). Transforming precision medicine: the potential of the clinical artificial intelligent single-cell framework. Clin. Transl. Med. 15 (1), e70096. 10.1002/ctm2.70096 39763060 PMC11705532

[B8] Bentley E. R. Little S. R. (2021). Local delivery strategies to restore immune homeostasis in the context of inflammation. Adv. Drug Deliv. Rev. 178, 113971. 10.1016/j.addr.2021.113971 34530013 PMC8556365

[B9] Bottinger K. Regl C. Schapertons V. Rapp E. Wohlschlager T. Huber C. G. (2024). Small is beautiful - examining reliable determination of low-abundant therapeutic antibody glycovariants. J. Pharm. Anal. 14 (10), 100982. 10.1016/j.jpha.2024.100982 39850237 PMC11755342

[B10] Cai L. Li Y. Tan J. Xu L. Li Y. (2023a). Targeting LAG-3, TIM-3, and TIGIT for cancer immunotherapy. J. Hematol. Oncol. 16 (1), 101. 10.1186/s13045-023-01499-1 37670328 PMC10478462

[B11] Cai L. Ke M. Wang H. Wu W. Lin R. Huang P. (2023b). Physiologically based pharmacokinetic model combined with reverse dose method to study the nephrotoxic tolerance dose of tacrolimus. Arch. Toxicol. 97 (10), 2659–2673. 10.1007/s00204-023-03576-3 37572130

[B12] Capelletti S. Garcia Soto S. C. Goncalves M. (2024). On RNA-Programmable gene modulation as a versatile set of principles targeting muscular dystrophies. Mol. Ther. 32 (11), 3793–3807. 10.1016/j.ymthe.2024.08.016 39169620 PMC11573585

[B13] Che Y. Chen Y. Wang Z. Zheng S. Xing K. Yuan S. (2022). The combination of rhodosin and MMF prolongs cardiac allograft survival by inhibiting DC maturation by promoting mitochondrial fusion. Oxid. Med. Cell Longev. 2022, 7260305. 10.1155/2022/7260305 35855862 PMC9288296

[B14] Chen Y. Xu Z. Sun H. Ouyang X. Han Y. Yu H. (2023). Regulation of CD8(+) T memory and exhaustion by the mTOR signals. Cell Mol. Immunol. 20 (9), 1023–1039. 10.1038/s41423-023-01064-3 37582972 PMC10468538

[B15] Cheng H. Y. Anggelia M. R. Lin C. H. (2025). Unraveling the roles of macrophages in vascularized composite allotransplantation. Biomedicines 13 (6), 1425. 10.3390/biomedicines13061425 40564144 PMC12189788

[B16] Cheung P. K. McCormick C. Crawford B. E. Esko J. D. Tufaro F. Duncan G. (2001). Etiological point mutations in the hereditary multiple exostoses gene EXT1: a functional analysis of heparan sulfate polymerase activity. Am. J. Hum. Genet. 69 (1), 55–66. 10.1086/321278 11391482 PMC1226048

[B17] Cong X. Zhang Z. Li H. Yang Y. G. Zhang Y. Sun T. (2024). Nanocarriers for targeted drug delivery in the vascular system: focus on endothelium. J. Nanobiotechnology 22 (1), 620. 10.1186/s12951-024-02892-9 39396002 PMC11470712

[B18] Crossland R. E. Perutelli F. Bogunia-Kubik K. Mooney N. Milutin Gasperov N. Pucic-Bakovic M. (2020). Potential novel biomarkers in chronic graft-versus-host disease. Front. Immunol. 11, 602547. 10.3389/fimmu.2020.602547 33424849 PMC7786047

[B19] Cvijic T. Horvat M. Plahutnik J. Golob A. Marusic J. (2024). Multivariate quantitative analysis of glycan impact on IgG1 effector functions. MAbs 16 (1), 2430295. 10.1080/19420862.2024.2430295 39572418 PMC11587841

[B20] Del Bello A. Treiner E. (2023). Immune checkpoints in solid organ transplantation. Biol. (Basel) 12 (10), 1358. 10.3390/biology12101358 PMC10604300 37887068

[B21] Doenst T. Kirov H. Bagiella E. Scherag A. Omerovic E. (2025). Challenges of conventional and novel approaches to clinical trial designs in cardiovascular medicine. Eur. J. Cardiothorac. Surg. 67 (3), ezaf056. 10.1093/ejcts/ezaf056 39980154

[B22] Du M. Zhang S. Wang X. Liu C. Pan L. Chen X. (2024). Specific knockout of macrophage SHP2 promotes macrophage M2 polarization and alleviates renal ischemia-reperfusion injury. iScience 27 (3), 109048. 10.1016/j.isci.2024.109048 38464592 PMC10924133

[B23] Fang Y. Zhang Q. Lv C. Guo Y. He Y. Guo P. (2023). Mitochondrial fusion induced by transforming growth factor-beta1 serves as a switch that governs the metabolic reprogramming during differentiation of regulatory T cells. Redox Biol. 62, 102709. 10.1016/j.redox.2023.102709 37116255 PMC10165137

[B24] Galan M. Fernandez-Mendez L. Nunez V. Femenia-Muina M. Figuera-Belmonte P. Moya-Ruiz E. (2025). cDC1s promote atherosclerosis *via* local immunity and are targetable for therapy. Circ. Res. 137 (3), 400–416. 10.1161/CIRCRESAHA.124.325792 40444360 PMC12272919

[B25] Gammie J. S. Stukus D. R. Pham S. M. Hattler B. G. McGrath M. F. McCurry K. R. (1999). Effect of ischemic time on survival in clinical lung transplantation. Ann. Thorac. Surg. 68 (6):2015–2019. 10.1016/s0003-4975(99)00903-0 10616969

[B26] Geng Q. Xu Y. Hu Y. Wang L. Wang Y. Fan Z. (2024). Progress in the application of Organoids-On-A-Chip in diseases. Organogenesis 20 (1), 2386727. 10.1080/15476278.2024.2386727 39126669 PMC11318694

[B27] Giarraputo A. Metzger E. Brousaides N. Marcin J. Avillach C. Smith R. N. (2025). Single-cell spatial transcriptomics reveal intraglomerular cell activation and ligand-receptor relationships in chronic, active antibody mediated rejection. Kidney Int. 109, 196–210. 10.1016/j.kint.2025.08.042 41173191

[B28] Halder S. Lawrence M. C. Testa G. Periwal V. (2025). Donor-specific digital twin for living donor liver transplant recovery. Biol. Methods Protoc. 10 (1), bpaf037. 10.1093/biomethods/bpaf037 40486178 PMC12141195

[B29] Heeg M. Goldrath A. W. (2023). Insights into phenotypic and functional CD8(+) T(RM) heterogeneity. Immunol. Rev. 316 (1), 8–22. 10.1111/imr.13218 37191051 PMC10462388

[B30] Heeger P. S. Haro M. C. Jordan S. (2024). Translating B cell immunology to the treatment of antibody-mediated allograft rejection. Nat. Rev. Nephrol. 20 (4), 218–232. 10.1038/s41581-023-00791-0 38168662

[B31] Horvath S. Raj K. (2018). DNA methylation-based biomarkers and the epigenetic clock theory of ageing. Nat. Rev. Genet. 19 (6), 371–384. 10.1038/s41576-018-0004-3 29643443

[B32] Hua Z. Zhou N. Zhou Z. Fu Z. Guo R. Akogo H. Y. (2025). Intranasal administration of stem cell derivatives for the treatment of AD animal models: a systematic review and meta-analysis. Stem Cell Res. Ther. 16 (1), 409. 10.1186/s13287-025-04555-4 40722027 PMC12306092

[B33] Islam M. M. Takeyama N. (2023). Role of neutrophil extracellular traps in health and disease pathophysiology: recent insights and advances. Int. Journal Molecular Sciences 24 (21), 15805. 10.3390/ijms242115805 37958788 PMC10649138

[B34] Iwase H. Ezzelarab M. B. Ekser B. Cooper D. K. (2014). The role of platelets in coagulation dysfunction in xenotransplantation, and therapeutic options. Xenotransplantation 21 (3), 201–220. 10.1111/xen.12085 24571124

[B35] Iyer A. A. Umadevi K. S. (2025). Design and analysis of TwinCardio framework to detect and monitor cardiovascular diseases using digital twin and deep neural network. Sci. Rep. 15 (1), 24376. 10.1038/s41598-025-08824-3 40628887 PMC12238484

[B36] James L. P. Kimberly R. Lindsell C. J. Meinzen-Derr J. K. O'Hara R. (2024). Scientia pro Bono humani generis: science for the benefit of humanity. J. Clin. Transl. Sci. 8 (1), e29. 10.1017/cts.2023.696 38384907 PMC10879989

[B37] Jaraba-Alvarez W. V. Uscanga-Palomeque A. C. Sanchez-Giraldo V. Madrid C. Ortega-Arellano H. Halpert K. (2025). Hypoxia-induced metabolic reprogramming in mesenchymal stem cells: unlocking the regenerative potential of secreted factors. Front. Cell Dev. Biol. 13, 1609082. 10.3389/fcell.2025.1609082 40552309 PMC12183037

[B38] Kammona O. Kiparissides C. (2020). Recent advances in antigen-specific immunotherapies for the treatment of multiple sclerosis. Brain Sci. 10 (6). 10.3390/brainsci10060333 32486045 PMC7348736

[B39] Karpen S. R. White J. K. Mullin A. P. O'Doherty I. Hudson L. D. Romero K. (2021). Effective data sharing as a conduit for advancing medical product development. Ther. Innov. Regul. Sci. 55 (3), 591–600. 10.1007/s43441-020-00255-8 33398663 PMC7780909

[B40] Kuppan P. Wong J. Kelly S. Lin J. Worton J. Castro C. (2023). Long-term survival and induction of operational tolerance to Murine Islet allografts by Co-Transplanting cyclosporine A microparticles and CTLA4-Ig. Pharmaceutics 15 (9), 2201. 10.3390/pharmaceutics15092201 37765170 PMC10537425

[B41] Kurd N. S. He Z. Louis T. L. Milner J. J. Omilusik K. D. Jin W. (2020). Early precursors and molecular determinants of tissue-resident memory CD8(+) T lymphocytes revealed by single-cell RNA sequencing. Sci. Immunol. 5 (47). 10.1126/sciimmunol.aaz6894 32414833 PMC7341730

[B42] Lee J. L. Linterman M. A. (2022). Mechanisms underpinning poor antibody responses to vaccines in ageing. Immunol. Lett. 241, 1–14. 10.1016/j.imlet.2021.11.001 34767859 PMC8765414

[B43] Li M. Yu J. Guo G. Shen H. (2023). Interactions between macrophages and biofilm during staphylococcus aureus-Associated implant infection: difficulties and solutions. J. Innate Immun. 15 (1), 499–515. 10.1159/000530385 37011602 PMC10315156

[B44] Li Z. Tian M. Yang Y. Wang Y. Zhang L. Huang F. (2025). Revealing the significance of tissue-resident memory T cells in lung adenocarcinoma through bioinformatic analysis and experimental validation. Front. Immunol. 16, 1600863. 10.3389/fimmu.2025.1600863 40642061 PMC12240778

[B45] Liu R. Zhao E. Yu H. Yuan C. Abbas M. N. Cui H. (2023). Methylation across the central dogma in health and diseases: new therapeutic strategies. Signal Transduct. Target Ther. 8 (1), 310. 10.1038/s41392-023-01528-y 37620312 PMC10449936

[B46] Liu Z. Liang Q. Ren Y. Guo C. Ge X. Wang L. (2023). Immunosenescence: molecular mechanisms and diseases. Signal Transduct. Target Ther. 8 (1), 200. 10.1038/s41392-023-01451-2 37179335 PMC10182360

[B47] Liu S. Cao Y. Cui K. Ren G. Zhao T. Wang X. (2024). Regulation of T helper cell differentiation by the interplay between histone modification and chromatin interaction. Immunity 57 (5), 987–1004 e1005. 10.1016/j.immuni.2024.03.018 38614090 PMC11096031

[B48] Lopez-Lopez V. Perez-Sanz F. de Torre-Minguela C. Marco-Abenza J. Robles-Campos R. Sanchez-Bueno F. (2021). Proteomics in liver transplantation: a systematic review. Front. Immunol. 12, 672829. 10.3389/fimmu.2021.672829 34381445 PMC8350337

[B49] Louis K. Fadakar P. Macedo C. Yamada M. Lucas M. Gu X. (2022). Concomitant loss of regulatory T and B cells is a distinguishing immune feature of antibody-mediated rejection in kidney transplantation. Kidney Int. 101 (5), 1003–1016. 10.1016/j.kint.2021.12.027 35090879 PMC9038633

[B50] Luo M. Samandi L. Z. Wang Z. Chen Z. J. Gao J. (2017). Synthetic nanovaccines for immunotherapy. J. Control Release 263, 200–210. 10.1016/j.jconrel.2017.03.033 28336379 PMC5603379

[B51] Murakami N. Borges T. J. Win T. S. Abarzua P. Tasigiorgos S. Kollar B. (2023). Low-dose interleukin-2 promotes immune regulation in face transplantation: a pilot study. Am. J. Transpl. 23 (4), 549–558. 10.1016/j.ajt.2023.01.016 PMC10318113 36740193

[B52] Niu D. Wei H. J. Lin L. George H. Wang T. Lee I. H. (2017). Inactivation of porcine endogenous retrovirus in pigs using CRISPR-Cas9. Science 357 (6357), 1303–1307. 10.1126/science.aan4187 28798043 PMC5813284

[B53] Nog R. Aggarwal Gupta C. Panza J. A. (2022). Role of MicroRNA in heart transplant. Cardiol. Rev. 30 (5), 253–257. 10.1097/CRD.0000000000000393 33883453

[B54] Nomura S. Ariyoshi Y. Watanabe H. Pomposelli T. Takeuchi K. Garcia G. (2020). Transgenic expression of human CD47 reduces phagocytosis of porcine endothelial cells and podocytes by baboon and human macrophages. Xenotransplantation 27 (1), e12549. 10.1111/xen.12549 31495971 PMC7007337

[B55] Palianina D. Di Roberto R. B. Castellanos-Rueda R. Schlatter F. Reddy S. T. Khanna N. (2023). A method for polyclonal antigen-specific T cell-targeted genome editing (TarGET) for adoptive cell transfer applications. Mol. Ther. Methods Clin. Dev. 30, 147–160. 10.1016/j.omtm.2023.06.007 37448595 PMC10336339

[B56] Prosser A. C. Kallies A. Lucas M. (2018). Tissue-Resident lymphocytes in solid organ transplantation: innocent passengers or the key to organ transplant survival? Transplantation 102 (3), 378–386. 10.1097/TP.0000000000002001 29135830

[B57] Randone P. Sanna E. Dolla C. Gallo E. Mingozzi S. Tarragoni R. (2024). Rescue with obinutuzumab and daratumumab as combined B cell/plasma cell targeting approach in severe posttransplant focal segmental glomerulosclerosis recurrence. Am. J. Transpl. 24 (10), 1896–1900. 10.1016/j.ajt.2024.06.010 39029875

[B58] Rao J. Wang Z. Yu F. Li J. Li W. Xuan Z. (2024). XBP1 facilitating NF-kappaB-p65 nuclear translocation promotes macrophage-originated sterile inflammation *via* regulating MT2 transcription in the ischemia/reperfusion liver. Cell Mol. Gastroenterol. Hepatol. 18 (6), 101402. 10.1016/j.jcmgh.2024.101402 39271015 PMC11546936

[B59] Rech Tondin A. Lanzoni G. (2025). Islet cell replacement and regeneration for type 1 diabetes: current developments and future prospects. BioDrugs 39 (2), 261–280. 10.1007/s40259-025-00703-7 39918671 PMC11906537

[B60] Reina-Campos M. Scharping N. E. Goldrath A. W. (2021). CD8(+) T cell metabolism in infection and cancer. Nat. Rev. Immunol. 21 (11), 718–738. 10.1038/s41577-021-00537-8 33981085 PMC8806153

[B61] Reichart B. Cooper D. K. C. Langin M. Tonjes R. R. Pierson R. N. Wolf E. (2023). Cardiac xenotransplantation: from concept to clinic. Cardiovasc Res. 118 (18), 3499–3516. 10.1093/cvr/cvac180 36461918 PMC9897693

[B62] Roggla G. Moser B. Roggla M. (1999). Seat space on airlines. Lancet 353 (9163), 1532. 10.1016/S0140-6736(05)67232-7 10232356

[B63] Sanna E. Nicassio S. R. Mella A. Mingozzi S. Manzione A. M. Dolla C. (2025). Targeting the alternative complement pathway by iptacopan abrogates C3 and strongly reduces C5b-9 deposition within glomeruli in IgA nephropathy recurrence after kidney transplantation. Am. J. Transpl. 25 (12), 2651–2657. 10.1016/j.ajt.2025.08.015 40854489

[B64] Schafer P. S. L. Dimitrov D. Villablanca E. J. Saez-Rodriguez J. (2024). Integrating single-cell multi-omics and prior biological knowledge for a functional characterization of the immune system. Nat. Immunol. 25 (3), 405–417. 10.1038/s41590-024-01768-2 38413722

[B65] Shang J. Hu S. Wang X. (2024). Targeting natural killer cells: from basic biology to clinical application in hematologic malignancies. Exp. Hematol. Oncol. 13 (1), 21. 10.1186/s40164-024-00481-y 38396050 PMC10885621

[B66] Shi W. Xu Y. Wei J. Zhang X. Zhu S. Guo H. (2025). Plant-derived secondary metabolites and nanotechnology: innovative strategies and emerging challenges in myocardial ischemia-reperfusion injury therapy. Front. Pharmacol. 16, 1529478. 10.3389/fphar.2025.1529478 40510425 PMC12159038

[B67] Shi Y. Zhang H. Miao C. (2025). Metabolic reprogram and T cell differentiation in inflammation: current evidence and future perspectives. Cell Death Discov. 11 (1), 123. 10.1038/s41420-025-02403-1 40155378 PMC11953409

[B68] Shu Y. Yang R. Wen H. Dong Q. Chen Z. Xiang Y. (2024). Myricetin reduces neutrophil extracellular trap release in a rat model of rheumatoid arthritis, which is associated with a decrease in disease severity. Innate Immun. 30 (2-4), 66–78. 10.1177/17534259241255439 38780369 PMC11165658

[B69] Singh A. K. Goerlich C. E. Shah A. M. Zhang T. Tatarov I. Ayares D. (2022). Cardiac xenotransplantation: progress in preclinical models and prospects for clinical translation. Transpl. Int. 35, 10171. 10.3389/ti.2022.10171 35401039 PMC8985160

[B70] Singh L. Nair L. Kumar D. Arora M. K. Bajaj S. Gadewar M. (2023). Hypoxia induced lactate acidosis modulates tumor microenvironment and lipid reprogramming to sustain the cancer cell survival. Front. Oncol. 13, 1034205. 10.3389/fonc.2023.1034205 36761981 PMC9906992

[B71] Singh A. K. Goerlich C. E. Zhang T. Lewis B. Hershfeld A. Braileanu G. (2025). Genetically engineered pig heart transplantation in non-human Primates. Commun. Med. (Lond) 5 (1), 6. 10.1038/s43856-025-00731-y 39774817 PMC11707197

[B72] Song M. Liu B. Wang H. Sun W. (2025). Lactate metabolism and lactylation modification: new opportunities and challenges in cardiovascular disease. MedComm (2020) 6 (7), e70269. 10.1002/mco2.70269 40599233 PMC12209596

[B73] Su L. Fang M. H. Zou J. Gao S. J. Gu X. Y. Meng X. D. (2021). Posttransplant blockade of CXCR4 improves leukemia complete remission rates and donor stem cell engraftment without aggravating GVHD. Cell Mol. Immunol. 18 (11), 2541–2553. 10.1038/s41423-021-00775-9 34635806 PMC8545944

[B74] Sun L. Su Y. Jiao A. Wang X. Zhang B. (2023). T cells in health and disease. Signal Transduct. Target Ther. 8 (1), 235. 10.1038/s41392-023-01471-y 37332039 PMC10277291

[B75] Tai Y. Li N. Fan J. Zhang H. Long H. Yan L. (2025). Characterization of peripheral immune cells in kidney transplantation recipients under different immunosuppressive treatments. Front. Immunol. 16, 1605664. 10.3389/fimmu.2025.1605664 40568581 PMC12187787

[B76] Tao Z. Luo Z. Zou Z. Ye W. Hao Y. Li X. (2025). Novel insights and an updated review of metabolic syndrome in immune-mediated organ transplant rejection. Front. Immunol. 16, 1580369. 10.3389/fimmu.2025.1580369 40330480 PMC12052740

[B77] Thim E. A. Kitelinger L. E. Rivera-Escalera F. Mathew A. S. Elliott M. R. Bullock T. N. J. (2024). Focused ultrasound ablation of melanoma with boiling histotripsy yields abscopal tumor control and antigen-dependent dendritic cell activation. Theranostics 14 (4), 1647–1661. 10.7150/thno.92089 38389838 PMC10879863

[B78] Tomusiak A. Floro A. Tiwari R. Riley R. Matsui H. Andrews N. (2024). Development of an epigenetic clock resistant to changes in immune cell composition. Commun. Biol. 7 (1), 934. 10.1038/s42003-024-06609-4 39095531 PMC11297166

[B79] Tran D. T. Tu Z. Alawieh A. Mulligan J. Esckilsen S. Quinn K. (2022). Modulating donor mitochondrial fusion/fission delivers immunoprotective effects in cardiac transplantation. Am. J. Transpl. 22 (2), 386–401. 10.1111/ajt.16882 PMC8813895 34714588

[B80] Troise D. Infante B. Mercuri S. Lindholm B. Kublickiene K. Stallone G. (2025). Exploring the immunological landscape of ischemia/reperfusion injury and graft rejection in kidney transplantation: shared mechanisms and insights. Cells 14 (18), 1443. 10.3390/cells14181443 41002408 PMC12468464

[B81] Trzupek D. Dunstan M. Cutler A. J. Lee M. Godfrey L. Jarvis L. (2020). Discovery of CD80 and CD86 as recent activation markers on regulatory T cells by protein-RNA single-cell analysis. Genome Med. 12 (1), 55. 10.1186/s13073-020-00756-z 32580776 PMC7315544

[B82] von Knethen A. Heinicke U. Weigert A. Zacharowski K. Brune B. (2020). Histone deacetylation inhibitors as modulators of regulatory T cells. Int. Journal Molecular Sciences 21 (7). 10.3390/ijms21072356 32235291 PMC7177531

[B83] Wagner J. C. Ronin E. Ho P. Peng Y. Tang Q. (2022). Anti-HLA-A2-CAR tregs prolong vascularized mouse heterotopic heart allograft survival. Am. J. Transpl. 22 (9), 2237–2245. 10.1111/ajt.17063 PMC9427704 35434896

[B84] Wang Y. Shi C. Guo J. Zhang D. Zhang Y. Zhang L. (2024). IDH1/MDH1 deacetylation promotes acute liver failure by regulating NETosis. Cell Mol. Biol. Lett. 29 (1), 8. 10.1186/s11658-023-00529-7 38172700 PMC10765752

[B85] Wang S. Wang Y. Li Z. Hong Y. Wang Z. Fan J. (2024). Early determination of potential critical quality attributes of therapeutic antibodies in developability studies through surface plasmon resonance-based relative binding activity assessment. MAbs 16 (1), 2374607. 10.1080/19420862.2024.2374607 38956880 PMC11225922

[B86] Wishart C. L. Spiteri A. G. Tan J. Counoupas C. Triccas J. A. Macia L. (2025). Deep metabolic profiling of immune cells by spectral flow cytometry-A comprehensive validation approach. iScience 28 (7), 112894. 10.1016/j.isci.2025.112894 40678547 PMC12268576

[B87] Wu E. Yin X. Liang F. Zhou X. Hu J. Yuan W. (2024). Analysis of immunogenic cell death in periodontitis based on scRNA-seq and bulk RNA-Seq data. Front. Immunol. 15, 1438998. 10.3389/fimmu.2024.1438998 39555084 PMC11568468

[B88] Xie C. Yang J. Gul A. Li Y. Zhang R. Yalikun M. (2024). Immunologic aspects of asthma: from molecular mechanisms to disease pathophysiology and clinical translation. Front. Immunol. 15, 1478624. 10.3389/fimmu.2024.1478624 39439788 PMC11494396

[B89] Xu X. Tao Y. Gao X. Zhang L. Li X. Zou W. (2016). A CRISPR-based approach for targeted DNA demethylation. Cell Discov. 2, 16009. 10.1038/celldisc.2016.9 27462456 PMC4853773

[B90] Xu Y. Wang J. Ren H. Dai H. Zhou Y. Ren X. (2023). Human endoderm stem cells reverse inflammation-related acute liver failure through cystatin SN-mediated inhibition of interferon signaling. Cell Res. 33 (2), 147–164. 10.1038/s41422-022-00760-5 36670290 PMC9892047

[B91] Xu J. Pu J. Chen H. Sun L. Fei S. Han Z. (2025). Role of microvascular pericyte dysfunction in antibody-mediated rejection following kidney transplantation. Ren. Fail 47 (1), 2458749. 10.1080/0886022X.2025.2458749 39910824 PMC11803764

[B92] Xu X. Pang Y. Fan X. (2025). Mitochondria in oxidative stress, inflammation and aging: from mechanisms to therapeutic advances. Signal Transduct. Target Ther. 10 (1), 190. 10.1038/s41392-025-02253-4 40500258 PMC12159213

[B93] Yang J. Lickliter J. D. Hillson J. L. Means G. D. Sanderson R. J. Carley K. (2021). First-in-human study of the safety, tolerability, pharmacokinetics, and pharmacodynamics of ALPN-101, a dual CD28/ICOS antagonist, in healthy adult subjects. Clin. Transl. Sci. 14 (4), 1314–1326. 10.1111/cts.12983 33503289 PMC8301585

[B94] Yi Z. Xi C. Menon M. C. Cravedi P. Tedla F. Soto A. (2024). A large-scale retrospective study enabled deep-learning based pathological assessment of frozen procurement kidney biopsies to predict graft loss and guide organ utilization. Kidney Int. 105 (2), 281–292. 10.1016/j.kint.2023.09.031 37923131 PMC10892475

[B95] Yokoyama A. P. H. Kutner J. M. de Moraes Mazetto Fonseca B. Mesquita G. Sakashita A. M. Dos Santos A. P. R. (2023). Neutrophil extracellular traps (NETs), transfusion requirements and clinical outcomes in orthotopic liver transplantation. J. Thromb. Thrombolysis 56 (2), 253–263. 10.1007/s11239-023-02825-7 37227652

[B96] Zhi Y. Li M. Lv G. (2023). Into the multi-omics era: progress of T cells profiling in the context of solid organ transplantation. Front. Immunol. 14, 1058296. 10.3389/fimmu.2023.1058296 36798139 PMC9927650

[B97] Zhang Q. Q. Zhang W. J. Chang S. (2023). HDAC6 inhibition: a significant potential regulator and therapeutic option to translate into clinical practice in renal transplantation. Front. Immunol. 14, 1168848. 10.3389/fimmu.2023.1168848 37545520 PMC10401441

[B98] Zhao B. Wei J. Jiang Z. Long Y. Xu Y. Jiang B. (2025). Mesenchymal stem cell-derived exosomes: an emerging therapeutic strategy for hepatic ischemia-reperfusion injury. Stem Cell Res. Ther. 16 (1), 178. 10.1186/s13287-025-04302-9 40229893 PMC11998454

[B99] Zhou Y. Chen Y. L. Huang X. Y. Chang Y. J. (2024). Desensitization strategies for donor-specific antibodies in HLA-mismatched stem cell transplantation recipients: what we know and what we do not know. Oncol. Ther. 12 (3), 375–394. 10.1007/s40487-024-00283-6 38879734 PMC11333671

